# Selectively-informed particle swarm optimization

**DOI:** 10.1038/srep09295

**Published:** 2015-03-19

**Authors:** Yang Gao, Wenbo Du, Gang Yan

**Affiliations:** 1 School of Electronic and Information Engineering, Beihang University, Beijing 100191, People's Republic of China; 2 Center for Complex Network Research and Department of Physics, Northeastern University, Boston, MA 02115 USA

## Abstract

Particle swarm optimization (PSO) is a nature-inspired algorithm that has shown outstanding performance in solving many realistic problems. In the original PSO and most of its variants all particles are treated equally, overlooking the impact of structural heterogeneity on individual behavior. Here we employ complex networks to represent the population structure of swarms and propose a selectively-informed PSO (SIPSO), in which the particles choose different learning strategies based on their connections: a densely-connected hub particle gets full information from all of its neighbors while a non-hub particle with few connections can only follow a single yet best-performed neighbor. Extensive numerical experiments on widely-used benchmark functions show that our SIPSO algorithm remarkably outperforms the PSO and its existing variants in success rate, solution quality, and convergence speed. We also explore the evolution process from a microscopic point of view, leading to the discovery of different roles that the particles play in optimization. The hub particles guide the optimization process towards correct directions while the non-hub particles maintain the necessary population diversity, resulting in the optimum overall performance of SIPSO. These findings deepen our understanding of swarm intelligence and may shed light on the underlying mechanism of information exchange in natural swarm and flocking behaviors.

## Introduction

Optimization^1^,^2^,^3^ aims to seek the minimal or maximal point in the constrained parameter space of a system, which is highly challenging due to the increasing complexity of real problems we face in modern society. To solve real-world optimization problems researchers learned from the collective behaviors of social animals, yielding several intelligent algorithms^4^,^5^,^6^. Among those, particle swarm optimization (PSO), proposed by Kennedy and Eberhart^5^, is a typical swarm-intelligence algorithm that derives the inspiration from the self-organization and adaptation in flocking phenomena^7^,^8^,^9^,^10^,^11^.

In PSO, a flock of particles move in a constrained parameter space, interact with each other, and update their velocities and positions according to their own and their neighbors' experiences, searching for the global optimum. Owing to its simplicity, effectiveness and low computational cost, PSO has gained significant popularity and improvements. Most studies on improving the PSO fall into three categories. (1) Modifying the model coefficients. Shi and Eberhart introduced an inertia weight to reduce the restriction on velocity and better control the scope of search^12^. Later on, they employed fuzzy system and stochastic mechanism to better adapt the inertia weight^13^. Clerc and Kennedy introduced a constriction coefficient to ensure the convergence of the particles^14^. Trelea used dynamical system theory to analyze the PSO algorithm and derived the guidelines for choosing appropriate parameters^15^. Zhan *et al.* proposed an adaptive PSO in which model coefficients can vary according to evolutionary states^16^. (2) Considering the population structure. Kennedy showed that the sociometric structure and small-world manipulation interacted with function can produce a significant effect on performance^17^. Kennedy and Mendes examined the impact of topological structure more detailedly, leading to the identification of superior population configurations^18^. Liu *et al.* proposed the scale-free PSO (SFPSO) which employs degree-heterogeneous (scale-free) topologies and is able to significantly improve the optimization performance^19^. (3) Altering the interaction modes. Mendes *et al.* revised the way how each particle is influenced by its neighbors, resulting in the fully-informed PSO (FIPSO)^20^,^21^ in which each particle learns from every individual in its neighborhood rather than the single best one. The performance of FIPSO is closely related to the population structure^22^. Liang *et al.* proposed the comprehensive learning PSO that allows each dimension of a particle to learn from different neighbors^23^. Li *et al.* proposed the adaptive learning PSO in which each particle can adaptively guide its behavior of exploration and exploitation^24^. They further proposed the self-learning PSO (SLPSO) that allows each particle to adaptively choose one of four learning strategies in different situations with respect to convergence, exploitation, exploration, and jumping out of the basins of attraction of local optima^25^.

However, most of the existing PSO algorithms treat all particles equally, prompting us to explore the impact of heterogeneous sight ranges: the hub particles (leaders) have a broad sight of the population; each non-hub particle (follower) has only a single source of information. The former would make the optimization process well guided by the leaders while the latter allows the followers to move without unnecessary interference. We found that our algorithm, selectively-informed PSO (SIPSO), taking into account the individuals' heterogeneity, can balance the exploration and the exploitation in the optimization process thus it achieves better performance.

In the following we will briefly introduce the PSO and its typical variants and then describe our SIPSO algorithm in detail.

### GPSO & LPSO

**Figure 1:**
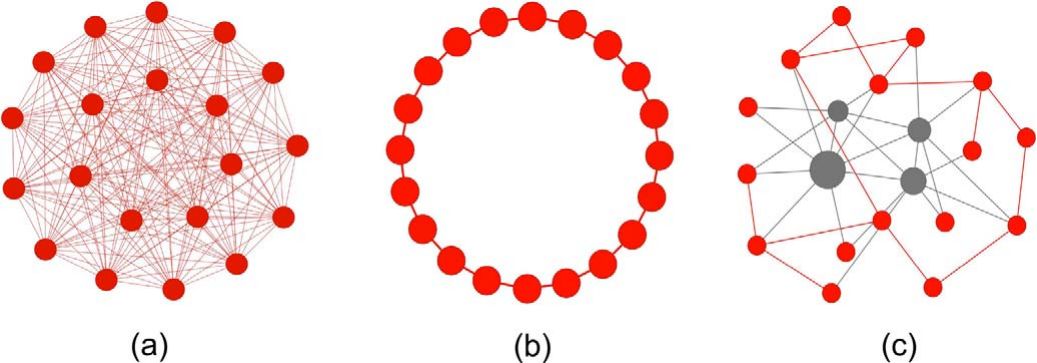
Network structure representing the interactions between particles. (a) A complete network with 20 nodes (particles). Each node connects to all others. (b) A ring network with 20 nodes. Each node links to its nearest two neighbors. (c) A scale-free network with 20 nodes, in which the node size represents the node degree, i.e. the number of edges associated with the node. It shows that most nodes have low degrees, yet there exist a few high-degree nodes (hubs).

For a minimum optimization problem with *D* independent variables and an objective function *f*(***x***), the PSO algorithm represents the potential solutions with a flock of particles. Each particle *i* has a position ***x****_i_* = [*x_i_*_1_, *x_i_*_2_, …*x_iD_*] and a velocity ***v****_i_* = [*v_i_*_1_, *v_i_*_2_, … *v_iD_*] in the *D*-dimensional space. The goal is to find an optimal position ***x****_i_* of any particle *i* that makes the objective function *f*(***x***) minimum. Initially the particles' positions and velocities are generated randomly. Then, at each time step (iteration), each particle updates its position and velocity according to the following equations^5^:
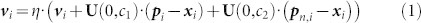

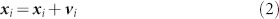
where 
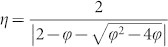
, *φ* = *c*_1_ + *c*_2_ > 4. Here ***p****_i_* is the best historical position found by particle *i*, ***p****_n_*_,*i*_ is the best historical position found by *i*'s neighbors, *c*_1_ and *c*_2_ are the acceleration coefficients. **U**(*a*, *b*) is a random number drawn at each iteration from the uniform distribution [*a*, *b*]. Therefore, *c*_1_ and *c*_2_ balance the impacts of each particle's own and its neighbors' experiences, and *η* indicates the learning rate. Based on previous extensive analysis^14^ we choose the appropriate settings as *c*_1_ = *c*_2_ = 2.05 and *η* = 0.7298. Previous studies^17^,^18^,^20^,^21^,^22^ have found that the interaction topology of particles has a great influence on final optimization results. Two versions of canonical PSO algorithm with different topologies are most commonly used: the GPSO with a fully connected network (Fig. 1(a)) and the LPSO with a ring (Fig. 1(b)). GPSO converges more rapidly than LPSO, yet, is more susceptible to be trapped at local optima^17^.

### FIPSO

In the canonical PSO each particle is influenced by itself and the best-performed particle in its neighborhood. This “single-informed” strategy may ignore some important information from the remaining neighbors. Mendes *et. al.* hence proposed a “fully-informed” version of PSO (FIPSO)^20^,^21^, in which each particle adjusts its velocity according to the experiences of its all neighbors:
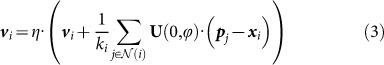
where 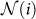 is the node set of *i*'s neighbors, *k_i_* is the number of *i*'s neighbors (*i.e.*, *k_i_* is *i*'s degree and 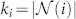), ***p****_j_* is the best historic position found by *j*. Studies^21^,^22^ have revealed that, with appropriate parameter settings, the FIPSO can outperform the traditional PSO, but it is susceptible to the topology alteration. In some topologies the FIPSO may perform even worse than the canonical PSO.

### SFPSO & SFIPSO

Recently, many natural and man-made networks have been found to exhibit scale-free property, *i.e.* the degree distribution is power-law^26^,^27^. Examples include neural networks^28^, citation networks^29^, World Wide Web^30^, Internet^31^, software engineering^32^, and on-line social networks^33^. In scale-free networks, only a few nodes are densely connected hubs and most nodes are low degree non-hub nodes, resulting in high heterogeneity of node's degrees (Fig. 1c). This discovery has triggered the interest of studying the impacts of underlying network structures on dynamical processes^34^,^35^,^36^,^37^,^38^,^39^,^40^ and also of introducing scale-free topologies into evolutionary optimization algorithms^19^,^41^,^42^,^43^. In particular, Liu *et al.* investigated the influence of scale-free population structure on the performance of PSO^19^. Their results indicated that the scale-free PSO (SFPSO) outperforms the traditional GPSO and LPSO. In the following we also compare our algorithm to the fully-informed versions of SFPSO and GPSO (called SFIPSO and GFIPSO hereafter, respectively).

### SLPSO

In most traditional PSO algorithms, a single learning mode is used for all particles, which may restrict the intelligence for a particular particle to deal with different situations. Li *et al.* proposed the self-learning PSO (SLPSO) that enables the particles to switch between four modes: exploitation, exploration, jumping out, and convergence^25^. Each mode has a set of operations to update the particles' velocity and position. A common strategy was introduced to allow each particle to adaptively choose the most suitable mode which depends on evolutionary stages and local fitness landscape. Experimental comparisons showed that SLPSO outperforms several peer algorithms in terms of mean value, success rate and overall ranking, especially for some complex high-dimensional functions. Yet, three key parameters of SLPSO need to be chosen very carefully through a parameter tuning approach, as these parameters significantly affect the algorithm's performance. Note that in SLPSO, although each particle is able to switch between different modes, the learning strategy of choosing suitable modes is identical for all particles.

### Selectively-informed PSO

The algorithms described above assumed that all particles are single-informed or fully informed, or adopt the same strategy for switching between different modes, overlooking the heterogeneity of individuals. Here we propose the selectively-informed PSO (SIPSO) algorithm that takes into consideration the heterogeneity of individuals' learning strategies. The population structure of our SIPSO is represented by a scale-free network (see **Methods**). And the learning strategy of each particle depends on its degree:
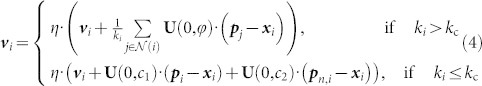
where *k_i_* is the degree of particle *i*, *k*_c_ is the threshold to determine a particle fully- or single-informed. The densely-connected hubs (*k* > *k*_c_) are provided with more information to better lead the optimization process. The non-hub particles (*k* ≤ *k*_c_) are less affected so that they can move in the search space with more freedom, maintaining the diversity of the population. Note that, when *k*_c_ = *k_min_* − 1, all the particles are fully-informed thus the algorithm is degenerated to SFIPSO; when *k*_c_ = *k_max_*, all the particles take the canonical learning strategy, turning the algorithm to SFPSO. Here we are interested in the information selectivity, i.e, *k_min_* − 1 < *k*_c_ < *k_max_*. For example, in Fig. 1c, when *k*_c_ = 5 the grey nodes (particles) with degree higher than 5 are fully-informed and the rest red nodes are single-informed.

## Results

### Overall performance

We test the performance of our algorithm on eight widely-used benchmark functions *f*_1–8_ (see **Methods**) and compare it to other seven algorithms for three criteria: *success rate*, *solution quality*, and *convergence speed* (see **Methods**). Note that in SIPSO the optimal value of the degree threshold *k*_c_ varies for different test functions. We also show the results for a fixed threshold (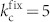) over all the functions.

Table 1 lists the comparison of success rate. Our algorithm SIPSO shows significant advantages, *i.e.*, 99% on *f*_8_ and 100% on all the other functions. Even with a fixed threshold 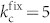 the SIPSO also gets very satisfactory success rates.

Table 2 lists the results in terms of solution quality. For each function, the best solutions are highlighted in bold and “–” means that the corresponding algorithm fails to reach the acceptable solution even once. For functions *f*_2–4_ our SIPSO remarkably outperforms the other algorithms, for *f*_1_, *f*_5_, *f*_6_ and *f*_8_ the SIPSO ranks 2*^nd^* of all the algorithms, while for *f*_7_ it ranks 3*^rd^*. When the degree threshold is fixed as 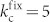, the solution quality still ranks top 3 of all the algorithms over eight test functions.

Table 3 shows the convergence speed of each algorithm, represented by the steps required to reach the goal value. Thus the smaller the number of required steps, the higher the convergence speed. The best cases are marked in bold. Our SIPSO has a relatively fast convergence speed on all the functions, ranking 2*^nd^* on *f*_1_, *f*_2_, *f*_3_, *f*_6_ and *f*_8_, 3*^rd^* on *f*_4_ and *f*_7_, 4*^th^* on *f*_5_. SFIPSO has the fastest convergence speed on *f*_1_, *f*_2_, *f*_3_, *f*_6_, and *f*_8_, and the GFIPSO converges fastest on *f*_4_, *f*_5_ and *f*_7_. It is worth noting that, faster convergence does not necessarily mean a better optimization trial. Actually, too fast convergence may lead to the problem of prematureness, *i.e.*, being trapped at local optima. For example, as shown in Table 2 the solution qualities of SFIPSO and GFIPSO are really bad for most benchmark functions, although their convergence are very fast. In the fully-informed algorithms, each particle's information can be quickly transferred to all other individuals in the swarm thus the algorithms converge rapidly, resulting in prematureness. In contrast, in our SIPSO, only the hub particles are fully-informed and there are many non-hub particles taking the single-informed learning strategy to maintain the population diversity. Consequently, our SIPSO can achieve better performance with a satisfactory convergence speed.

### The impact of *k*_c_

**Figure 2:**
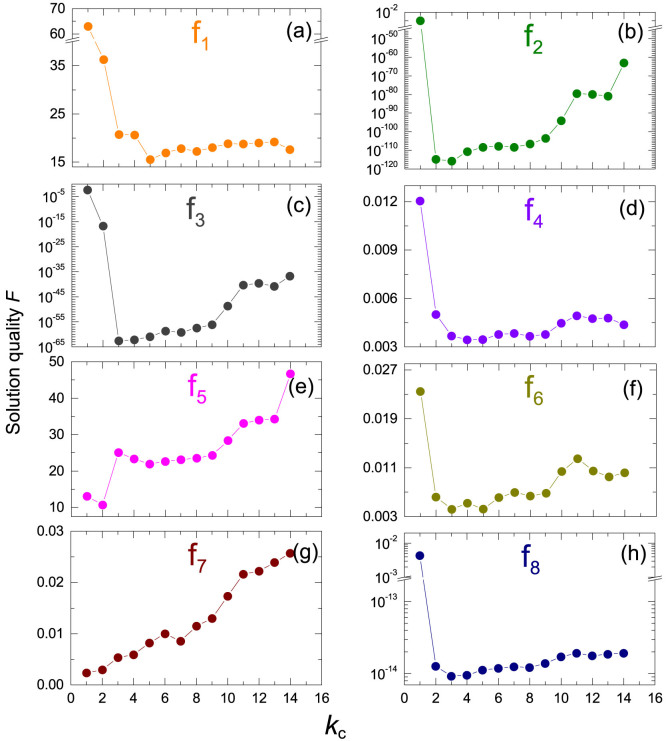
The impact of k_c_ on the solution quality of the algorithm for eight benchmark functions. In all sub-figures the vertical axes represent the solution quality for each function f by the average fitness of different runs 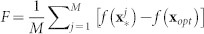, where M is the number of independent runs, 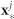 is the best solution found in j-th run, and **x**_opt_ is the (known) optimum solution for the given function f. Therefore, the smaller the value of F, the better the performance of the algorithm.

**Figure 3:**
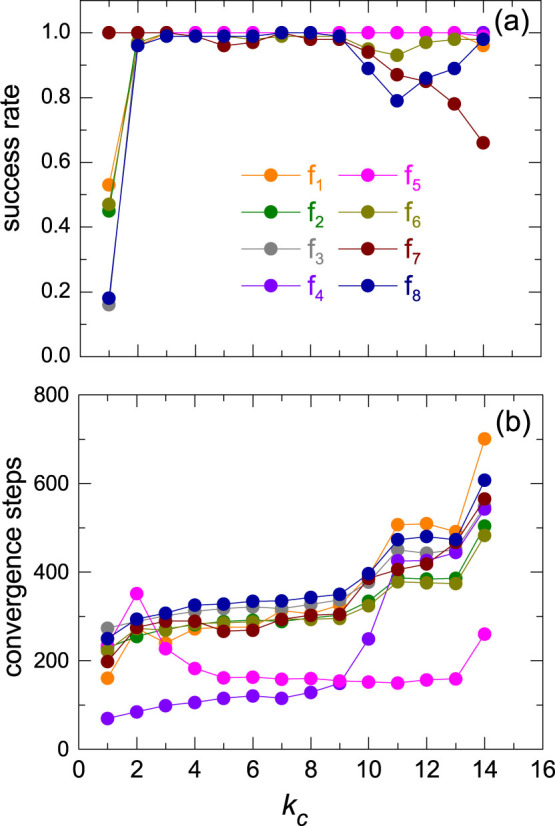
The impact of k_c_ on the success rate and convergence speed. (a) The success rate represented the proportion of successful runs. (b) The steps required to reach the goal value. The smaller the number of required steps, the higher the convergence speed.

As described above we find that for each function there is an optimal value of the threshold *k*_c_ with which our algorithm SIPSO performs best. Hence we investigate the impact of *k*_c_ on the performance for all eight benchmark functions. The results of solution quality, success rate and convergence speed are shown in Figs. 2 and 3. One can see that, for the solution quality on all functions except *f*_5_ and *f*_7_ SFPSO (the rightmost data point) outperforms SFIPSO (the leftmost data point), while for *f*_5_ and *f*_7_ it reverses. However, on all the functions except for *f*_7_, neither SFIPSO nor SFPSO is able to obtain the best result. With *k*_c_ between *k_min_* and *k_max_* our algorithm SIPSO achieves the best performance (Fig. 2). Similar results for success rate are shown in Fig. 3(a). Our SIPSO has high success rate on all functions with an appropriate *k*_c_. As shown in Fig. 3(b), increasing the number of fully-informed particles can significantly improve the convergence speed and our SIPSO has moderate speed of convergence.

### The microscopic point of view

To uncover the underlying mechanism of our algorithm, we explore the optimization process from a microscopic point of view. We compare our SIPSO (*k_min_* − 1 < *k*_c_ < *k_max_*) to SFIPSO (*k*_c_ = *k_min_* − 1) and SFPSO (*k*_c_ = *k_max_*) that are all on scale-free networks, excluding the influence of other factors. For the sake of simplicity, in the following we will present the results for the function *f*_1_. The results for other functions are alike and not shown here.

**Figure 4:**
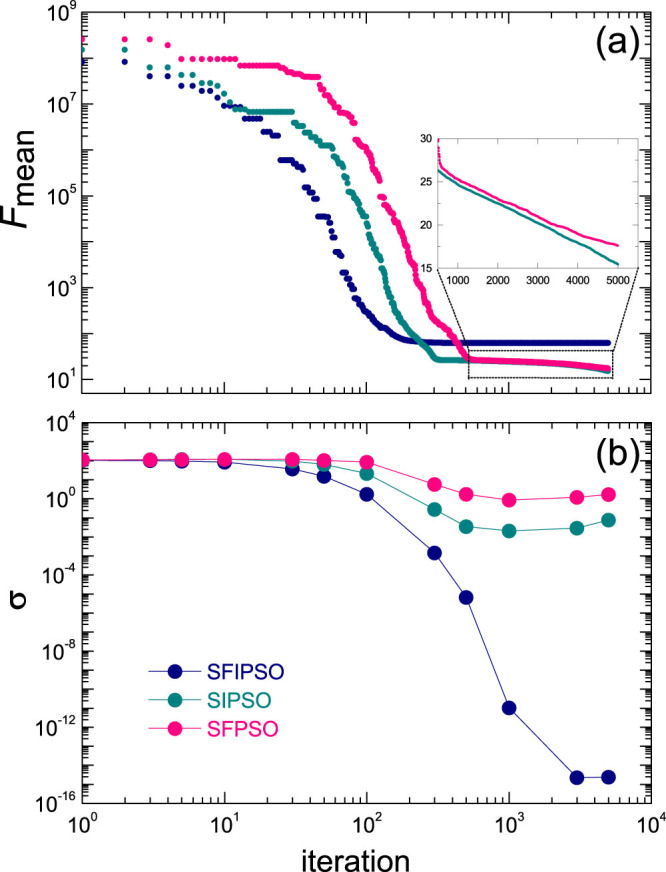
The mean fitness and the diversity of the swarm population during the optimization process. (a) The evolution of the mean fitness on the function f_1_, i.e., 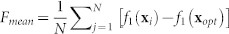 where N is the total number of particles, **x**_i_ is the position of particle i, and **x**_opt_ = **1** is the optimum solution of f_1_. The inset shows the last steps for SIPSO and SFPSO. (b) The evolution of the population diversity σ (see main text) during the optimization processes.

First, we examine the mean fitness (*F_mean_*) of the swarm population during an optimization process, with the definition 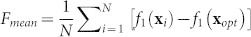 where *N* is the total number of particles, **x***_i_* is the position of particle *i*, and **x***_opt_* = **1** is the optimum solution of *f*_1_. As shown in Fig. 4(a) the SFIPSO has the fastest convergence as each particle uses full information from all of its neighbors, but it is trapped at some local optima in the early stage (~ 150 iterations). Despite their relatively low convergence SIPSO and SFPSO are able to achieve higher qualities of final solutions, and SIPSO is the best for the mean fitness.

Second, we compare the population diversity of SFPSO, SFIPSO and SIPSO, which indicates the extent of exploration during the searching process of the swarm. The population diversity is defined as^45^ 
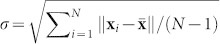
 where *N* is the total number of particles, and 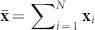 is the mean position (center) of the swarm. Thus, the larger the *σ*, more diverse is the swarm. And a very small *σ* means that all particles are aggregated together, diminishing the capability of exploration. As shown in Fig. 4(b), the diversity of SFIPSO decreases quickly to a very small value due to the information redundancy of the fully-informed learning. Consequently, SFIPSO is not able to escape once gets stuck at a local optimum. Both SFPSO and SIPSO have a high level of diversity during the optimization, which ensure the thorough search in the parameter space thus improve the probability of finding the global optimum.

**Figure 5:**
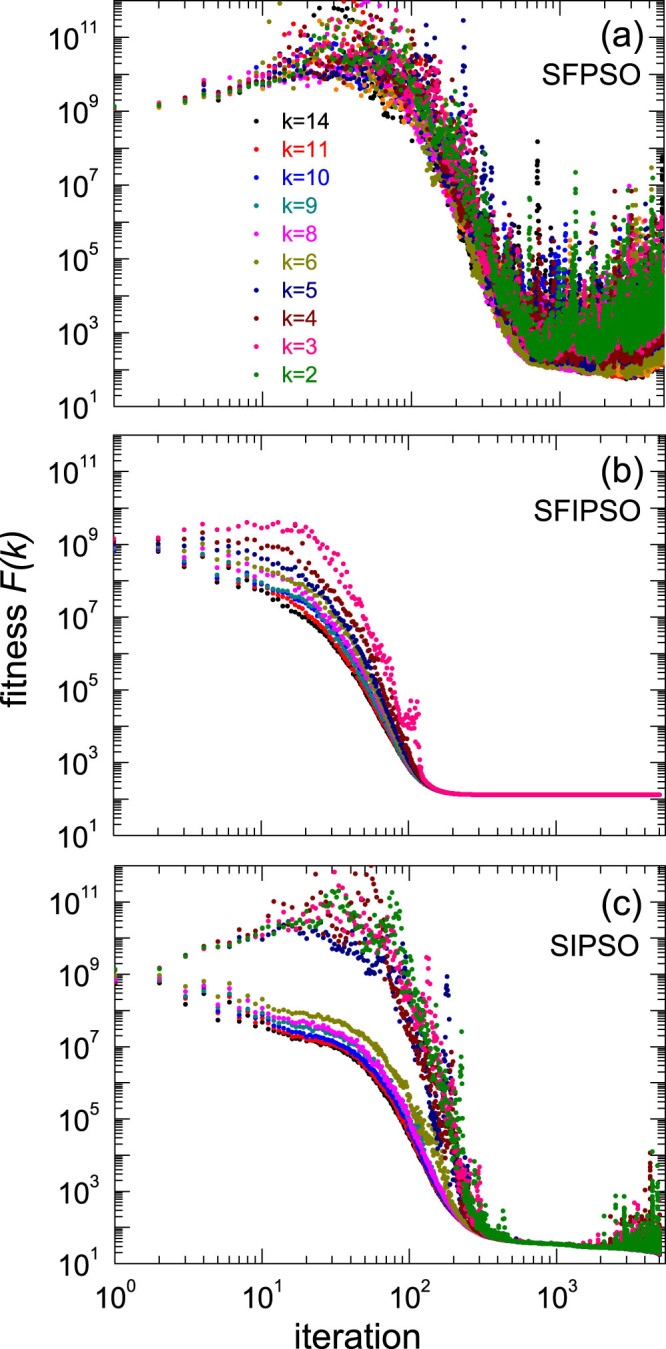
The fitness of particles with different degrees during the optimization process. Here 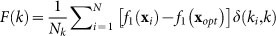, where N_k_ is the number of particles with degree k. And δ(k_i_, k) = 1 if k_i_ = k, and 0 otherwise. (a) SFPSO, i.e. k_c_ = k_max_; (b)SFIPSO, i.e. k_c_ = k_min_ − 1. (c) SIPSO, i.e. k_min_ − 1 < k_c_ < k_max_, here we show the evolution of F(k) for k_c_ = 5.

Furthermore, we investigate the fitness of particles with different degrees, *i.e.*, 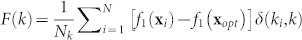, where *N_k_* is the number of particles with degree *k*. *δ*(*k_i_*, *k*) = 1 if *k_i_* = *k*, and 0 otherwise. The particles in SFPSO have only one information source, which is very unstable during the optimization process. So the fluctuation of the particles' fitness in SFPSO are violent (Fig. 5(a)). In SFIPSO, all particles are fully-informed, making the algorithm converge fast but prematurely (Fig. 5(b)). Our SIPSO combines the advantages of the two algorithms. The fitness of hub particles monotonously decreases, indicating that the hubs play the role of guiding the swarm. On the contrary, the non-hub particles have oscillating fitness, maintaining the necessary diversity of the swarm (Fig. 5(c)). The two different roles of the particles in SIPSO result in the appropriate trade-off between the convergence speed and the population diversity.

## Discussion

Taking into account the heterogeneity of individuals behaviors in flocking we propose the Selectively-Informed Particle Swarm Optimization (SIPSO) algorithm. In SIPSO, the particles interact with their neighbors and change the searching direction and speed by learning from the experiences of themselves and their neighbors. Each particle's learning strategy depends on its degree: the hubs are able to learn from all of their neighbors (fully-informed) while each non-hub particle learns from a single yet best-performed neighbor. Consequently, the hubs have bird's eye views of the swarm and can better lead the population; the non-hub particles are less influenced thus can search in the space with high freedom, maintaining the diversity of the population.

We test the performance of our SIPSO on eight benchmark functions. The results show that SIPSO has high success rate, high solution quality, and acceptable convergence speed. We examine the optimization process from a microscopic point of view and reveal that, indeed, there are two different roles that the particles play in the SIPSO. Moreover, our algorithm is able to balance the population diversity and the convergence speed during optimization processes, improving the overall performance in comparison with other seven algorithms.

It is worth noting that we do not introduce adaptation into our SIPSO algorithm, i.e., all parameters including *k_c_* are set initially and do not change during the optimization process, but instead we discriminate the nodes with different degrees, in contrast to SLPSO which adopts adaptive strategies in search of the optimum. Despite the lack of adaptation, our SIPSO works very well in the benchmark test functions. This finding uncovers the importance of considering the individuals' heterogeneity in particle swarm optimization. Nevertheless, as shown in previous works (e.g., refs. 24, 25), adaptation can improve PSO's performance. It is fairly expected that adaptively tuning the value of *k_c_* during the searching process could improve our SIPSO's performance, which deserves future pursuits.

## Methods

### Benchmark functions

To make a comprehensive comparison to test the effectiveness of our algorithm we designed extensive experiments. We choose eight benchmark functions (Table 4) that have been widely used^17^,^18^,^20^,^21^,^44^. Functions *f*_1_ − *f*_4_ are unimodal, which are relatively easy to solve. Functions *f*_5_ − *f*_8_ are multi-modal with a large number of local optima so that the algorithm really suffers from being premature. Functions *f*_6_ and *f*_7_ are the same Griewank function with different dimensions. In fact, *f*_7_ is considered more difficult^18^. Column 2 shows the formula of the fitness function. Column 3 shows the dimension of the problem *D*. Column 4 gives the range that variables can take. In column 5 the optimum values of the problems are presented. Column 6 defines the goal value to judge whether a run (trial) is successful or not.

### Parameter settings

The parameters of experiments are set as follows. The population size is 50. For each algorithm and each benchmark function, the experiment consists of 100 independent runs. The maximal iteration is 5000. For SFPSO, SFIPSO and SIPSO, the scale-free network has maximal degree 14 and minimal degree 2. We generate the scale-free networks by Barabási-Albert model^46^, which has two main mechanisms: growth and preferential attachment. Starting with *m*_0_ fully-connected nodes, at each time step we add a new node to the network and connect it to *m* existing nodes(*m* < *m*_0_). The probability *P_i_* that the new node is connected to an existing node *i* depends on *i*'s degree: 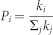, where *j* runs over all the existing nodes. Here we set the parameters *m*_0_ = 4 and *m* = 2.

### Criteria

To compare the performance of different algorithms we use three criteria: solution quality, convergence speed, and success rate. The solution quality is the final fitness value at the end of 5000 iterations. The convergence speed is represented by the number of iterations required to reach the goal. Obviously, the larger the number of required iterations, the lower the convergence speed. The success rate is the fraction of successful runs. Both the solution quality and the convergence speed are average values over the successful runs.

## Author Contributions

Y.G., W.B.D. and G.Y. designed and performed the research, analyzed the results, and wrote the paper.

## Figures and Tables

**Table 1:** *Success rate*

	*f*_1_	*f*_2_	*f*_3_	*f*_4_	*f*_5_	*f*_6_	*f*_7_	*f*_8_
GPSO	0.99	1.00	1.00	1.00	1.00	0.87	0.34	0.25
LPSO	0.92	1.00	1.00	1.00	1.00	1.00	0.71	1.00
SFPSO	0.96	1.00	1.00	1.00	1.00	0.99	0.66	0.98
GFIPSO	0	0	0	0.74	0.52	0	0.98	0
LFIPSO	0.99	1.00	1.00	1.00	0.88	1.00	1.00	1.00
SFIPSO	0.53	0.45	0.16	1.00	1.00	0.47	1.00	0.18
SLPSO	0.99	1.00	0.16	0.10	1.00	0.96	0.66	1.00
SIPSO	1.00 (*k*_c_ = 5)	1.00 (*k*_c_ = 3)	1.00 (*k*_c_ = 3)	1.00 (*k*_c_ = 4)	1.00 (*k*_c_ = 2)	1.00 (*k*_c_ = 3)	1.00 (*k*_c_ = 2)	0.99 (*k*_c_ = 3)
SIPSO ( 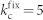 )	1.00	1.00	1.00	1.00	1.00	0.99	0.96	0.99

**Table 2:** *Solution quality*

	*f*_1_	*f*_2_	*f*_3_	*f*_4_	*f*_5_	*f*_6_	*f*_7_	*f*_8_
GPSO	1.96*e* + 1	3.00*e* − 100	6.78*e* − 23	4.12*e* − 3	6.01*e* + 1	9.96*e* − 3	3.55*e* − 2	1.24*e* − 14
LPSO	2.31*e* + 1	8.60*e* − 47	7.90*e* − 27	9.46*e* − 3	5.65*e* + 1	5.56*e* − 3	2.80*e* − 2	**7.69*e*** − **15**
SFPSO	1.76*e* + 1	8.61*e* − 64	1.99*e* − 37	4.37*e* − 3	4.67*e* + 1	1.02*e* − 2	2.57*e* − 2	1.93*e* − 14
GFIPSO	-	-	-	2.94*e* − 2	5.71*e* + 1	-	**1.41*e*** − **3**	-
LFIPSO	2.59*e* + 1	3.7*e* − 11	7.03*e* − 7	5.60*e* − 3	8.32*e* + 1	**1.96*e*** − **5**	8.87*e* − 3	2.96*e* − 6
SFIPSO	6.30*e* + 1	2.54*e* − 3	4.03*e* − 3	1.21*e* − 2	1.31*e* + 1	2.35*e* − 2	2.34*e* − 3	4.35*e* − 3
SLPSO	**2.28*e*** − **2**	1.14*e* − 58	1.20*e* − 33	4.39*e* − 2	**0**	1.66*e* − 2	3.97*e* − 2	2.34*e* − 14
SIPSO	1.55*e* + 1 (*k*_c_ = 5)	**1.64*e*** − **116** (*k*_c_ = 3)	**2.86*e*** − **63** (*k*_c_ = 3)	**3.45*e*** − **3** (*k*_c_ = 4)	1.07*e* + 1 (*k*_c_ = 2)	4.23*e* − 3 (*k*_c_ = 3)	2.94*e* − 3 (*k*_c_ = 2)	9.24*e* − 15 (*k*_c_ = 3)
SIPSO ( 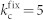 )	1.55*e* + 1	4.67 − 109	1.20*e* − 61	3.45*e* − 3	2.19*e* + 1	4.25*e* − 3	8.21*e* − 3	1.12*e* − 14

**Table 3:** *Convergence speed*

	*f*_1_	*f*_2_	*f*_3_	*f*_4_	*f*_5_	*f*_6_	*f*_7_	*f*_8_
GPSO	524.5	314.5	485.2	358.0	162.7	300.0	484.5	400.2
LPSO	856.4	660.0	764.8	1006.1	388.0	646.2	648.9	887.3
SFPSO	700.7	503.4	548.5	541.9	260.6	482.5	564.8	607.2
GFIPSO	-	-	-	**23.4**	**31.7**	-	**47.2**	-
LFIPSO	1985.1	2209.1	2594.9	1209.6	3367.2	2238.5	1773.0	2512.1
SFIPSO	**160.6**	**229.4**	**274**	70.0	234.7	**222.9**	198.1	**250.2**
SLPSO	854.2	1798.3	1851.7	4836.0	1762.1	1730.5	1146.1	1916.4
SIPSO	275.1 (*k*_c_ = 5)	271.2 (*k*_c_ = 3)	300.9 (*k*_c_ = 3)	105.6 (*k*_c_ = 4)	351.7 (*k*_c_ = 2)	269.2 (*k*_c_ = 3)	275.7 (*k*_c_ = 2)	307.8 (*k*_c_ = 3)
SIPSO ( 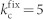 )	275.1	288.5	317.3	115.0	161.9	285.7	266.6	327.6

**Table 4:** *Benchmark functions*

Name	Formula	*D*	Range	Optimum	Goal
Rosenbrock	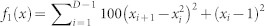	30	[−30, 30]*^D^*	0	100
Sphere	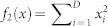	30	[−100, 100]*^D^*	0	0.01
Schwefel's P2.22	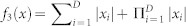	30	[−10, 10]*^D^*	0	0.01
QuadricNoise	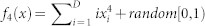	30	[−1.28, 1.28]*^D^*	0	0.05
Rastrigin	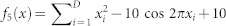	30	[−5.12, 5.12]*^D^*	0	100
Griwank	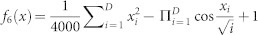	30	[−600, 600]*^D^*	0	0.05
Griwank	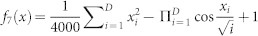	10	[−600, 600]*^D^*	0	0.05
Ackley	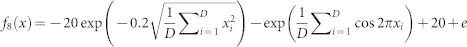	30	[−32, 32]*^D^*	0	0.01
